# Acid-free glyoxal as a substitute of formalin for structural and molecular preservation in tissue samples

**DOI:** 10.1371/journal.pone.0182965

**Published:** 2017-08-10

**Authors:** Gianni Bussolati, Laura Annaratone, Enrico Berrino, Umberto Miglio, Mara Panero, Marco Cupo, Patrizia Gugliotta, Tiziana Venesio, Anna Sapino, Caterina Marchiò

**Affiliations:** 1 Department of Medical Sciences, University of Turin, Turin, Italy; 2 Candiolo Cancer Institute - Fondazione del Piemonte per l'Oncologia (FPO), IRCCS, Candiolo, Italy; 3 Pathology Division, Azienda Ospedaliera Universitaria Città della Salute e della Scienza di Torino, Turin, Italy; Universidade de Mogi das Cruzes, BRAZIL

## Abstract

Tissue fixation in phosphate buffered formalin (PBF) remains the standard procedure in histopathology, since it results in an optimal structural, antigenic and molecular preservation that justifies the pivotal role presently played by diagnoses on PBF-fixed tissues in precision medicine. However, toxicity of formaldehyde causes an environmental concern and may demand substitution of this reagent. Having observed that the reported drawbacks of commercially available glyoxal substitutes of PBF (Prefer, Glyo-fix, Histo-Fix, Histo-CHOICE, and Safe-Fix II) are likely related to their acidity, we have devised a neutral fixative, obtained by removing acids from the dialdehyde glyoxal with an ion-exchange resin. The resulting glyoxal acid-free (GAF) fixative has been tested in a cohort of 30 specimens including colon (N = 25) and stomach (N = 5) cancers. Our results show that GAF fixation produces a tissue and cellular preservation similar to that produced by PBF. Comparable immuno-histochemical and molecular (DNA and RNA) analytical data were obtained. We observed a significant enrichment of longer DNA fragment size in GAF-fixed compared to PBF-fixed samples. Adoption of GAF as a non-toxic histological fixative of choice would require a process of validation, but the present data suggest that it represents a reliable candidate.

## Introduction

Fixation of histological specimens in formalin is in practice since over a century [1, 2] and still represents the procedure of choice for tissue preservation [3]. Over time, additional and ancillary techniques such as immunohistochemistry (IHC) and molecular analyses have been optimized in formalin-fixed paraffin embedded (FFPE) tissues, so that a sudden change of fixative is presently considered as impractical being potentially detrimental to the quality of diagnostic pathology. On the other hand, environmental authorities are increasingly concerned for the objective toxicity of this volatile reagent, so that a banning of formalin from 2016 has been proposed in the European Community. This has been stated by the EC Regulation n.605/2014 of 05.06.2014 that modifies the EC Regulation n.1272/2008 defining formalin as a carcinogen (category 1B/2) and mutagen. This regulation may exert a heavy impact on diagnostic pathology. Reaction to this status of affairs is presently limited to adoption of protective procedures, designed to prevent excessive exposure to formaldehyde vapors.

To be accepted by the scientific community, an “alternative fixative” should likely be an aldehyde, thus acting in a chemical reaction similar to that of formalin and affecting proteins and nucleic acids in a comparable way. This would avoid dramatic effects on IHC and molecular procedures and permit their use with minor adjustments, so that the bulk of the acquired and internationally accepted diagnostic parameters would not be lost. In addition, a possible alternative fixative should be relatively cheap, ideally in line with formalin, so as not to increase the final cost of histopathologic examinations. Moreover, it should not be toxic to avoid further restrictions. Finally, it should be rather fast, without affecting present turn around times.

Glyoxal (aka ethanedial, oxalaldehyde) was proposed in 1943 [4] as a fixative alternative to formalin since it is a simple di-aldehyde. As reported by Harke & Höffler [5] glyoxal does not appear to evaporate from solution. Indeed, the reported Henry law constant of ≤3.38 × 10–4Pa·m3/mol [6] indicates that glyoxal is essentially non-volatile with regard to the aqueous phase. Glyoxal is not classifiable as a human carcinogen [7], nevertheless is irritating to skin and eyes [7]. Tumor-promoting activity of glyoxal has been reported in rats subjected to long-term exposure to this agent in drinking water [8].

Taken together these data show that glyoxal has a very low toxicity even though holding a similar reactivity to formaldehyde.

Several studies have described the effect of glyoxal on tissues [9–11] and a variety of fixatives based on this reagent have been proposed. Still, criticisms have been reported, discouraging the use of this fixative as an alternative to formalin [3, 12]. In particular, it has been claimed that glyoxal-fixed tissues show clarity of cellular details, erythrocytes are lysed and microcalcifications are dissolved [13]. In addition, fluorescence *in situ* hybridization (FISH) analysis led to technically-compromised results [14, 15] and extraction and sequencing nucleic acids proved unsatisfactory [12, 14, 16–18].

By taking these phenomena into account and having observed that commercially available glyoxal is strongly acid, we reasoned that this peculiar acidity may be responsible for the observed detrimental effect on tissues. Acidification of glyoxal is likely due to its fast oxidation that leads to formation of acids, mainly glyoxilic acid, a very strong acid [19].

In this study we aimed to assess whether an acid-free form of glyoxal could represent a novel tissue fixative by investigating reliability of morphological details, IHC reactions and molecular analyses in a series of surgical specimens, in which *ad hoc* parallel sampling was performed to allow formalin- and glyoxal-fixed samples.

## Materials and methods

### Reagents

Glyoxal (40% in water) and basic ion-exchange resins (Amberlite^®^ IRA-400 chloride form and Amberlite^®^ IRA-67) were all purchased from Sigma (Milan, Italy). Addition of commercial glyoxal (40% in water) to a 0.1 M phosphate buffer pH 7.3 at a final concentration of 2% resulted in a drop of pH to the acidic side and this solution was in the range of pH 4 after 6 months. To deprive glyoxal of acids and thus to obtain Glyoxal Acid-Free (GAF), 250 g of the strongly basic ion-exchange resin were moistened with de-ionized H_2_O, then activated by a short wash with NaOH 1 M. Following washes in water to remove sodium hydroxide, 150 ml of glyoxal were added. The resin was allowed to act for 30 min at RT and then removed by passages through a filter. The resulting, water-clear liquid, whose pH is around neutrality, represented 40% glyoxal deprived of acids. We adopted a 2% solution of GAF as the fixative of choice. The 2% solution was selected by referring to the same amount of aldehyde as present in 4% formalin.

The resin, once used to remove acids from glyoxal, can be re-generated (with a bath of 1M NaOH) and re-used.

A 2% GAF solution in 0.1 M phosphate buffer pH 7.3 is stable for a few weeks then gradually undergoes oxidation producing an acidic reagent with sub-optimal fixation properties. To overcome the stability problem, we produced a stock solution containing 20% GAF in 50% ethanol (Carlo Erba, Milan, Italy) added with 0.1 g insoluble calcium carbonate (Sigma-Aldrich) in 100 ml of the solution (stock solution). The final (working) solution employed as GAF fixative was obtained by diluting the stock solution (exempted of calcium carbonate) 1:10 in 0.11 M phosphate buffer pH 7.3.

### Human tissue samples

Thirty human surgical samples (25 colorectal adenocarcinomas and 5 gastric adenocarcinomas) harboring a lesion of adequate dimensions (>2 cm) to allow multiple sampling in parallel were sampled according to standard practice and fixed in parallel in PBF and in GAF (working solution).

Following overnight fixation at RT, dehydration in alcohol and paraffin embedding followed standard procedures to paraffin embedding with an automatic processor (Leica ASP 300, Leica Microsystems, Wetzlar, Germany). Sections were stained in Haematoxylin and Eosin (H&E). Samples were subjected to i) immunohistochemical staining (whole cohort, 30 cases); ii) FISH analysis (five gastric carcinomas); iii) molecular analyses (eight cases of colorectal cancer).

The study was approved by the Ethic Institutional Review Board (IRB) responsible for "Biobanking and use of human tissues for experimental studies"—Department of Medical Sciences, University of Turin.

### Immunohistochemistry

Three μm thick sections were cut from tissue blocks and IHC was performed using an automated platform (Ventana BenchMark AutoStainer, Ventana Medical Systems, USA). Positive and negative controls (omission of the primary antibody and IgG-matched serum) were included for each immunohistochemical run. Optimized IHC conditions, assessed following experimental trials, are reported in Table 1. Antigen Retrieval was performed with Cell Conditioning Solution 1 and 2 (CC1 and CC2, both from Ventana Medical Systems, Inc.). CC1 is a tris based buffer with a slightly basic pH, while CC2 is a citrate buffer at a slightly acidic pH. Both solutions work at a controlled temperature of 95°C. We also tested a Buffer pH 8.6 and heat-induced epitope retrieval at 125°C in pressure cooker [20].

**Table 1:** Antibodies and antigen retrieval methods used for immunohistochemical reactions. Ab: antibody; CC1/CC2: cell conditioning Ventana Apparatus; CEA: carcinoembryonic antigen; CGA: chromogranin; CK: cytokeratin; F: formalin fixation; G: glyoxal fixation; SMA: smooth muscle actin; CAD-E: E-cadherin; PHH3: phospho-histone H3; TTF1: thyroid transcription factor 1.

Antibody	Clone	Species	Manufacturer	Dilution	Antigen Retrieval	Primary Ab Incubation
**HER2**	4b5	Rabbit	Roche	Prediluted	CC1, 36min	20min
**Ki67**	MIB-1	Mouse	Dako	1:50	CC1, 36min (F) CC2, 60min (G)	20min
**CK20**	SP33	Rabbit	Roche	Prediluted	CC1, 36min	20min
**PanCK**	AE1/AE3/PCK26	Mouse	Roche	Prediluted	Protease 1, 4min	20min
**SMA**	1A4	Mouse	Roche	Prediluted	CC1, 8min	20min
**CEA**	TF 3H8-1	Mouse	Roche	Prediluted	CC1, 8min	20min
**S100**	polyclonal	Rabbit	Roche	Prediluted	No treatment	20min
**CGA**	LK2H10	Mouse	Roche	Prediluted	No treatment	20min
**CDX2**	EPR2764Y	Rabbit	Roche	Prediluted	CC1, 60min (F) CC1, 92min (G)	20min
**TTF1**	8G7G3/1	Rabbit	Roche	Prediluted	CC1, 36min (F) CC1, 90min (G)	24min
**PHH3**	polyclonal	Rabbit	Roche	Prediluted	CC1, 36min (F) CC1, 60min (G)	32min
**CAD-E**	EP700Y	Mouse	Roche	Prediluted	Protease 1, 4min (F) CC1, 60min (G)	32min
**CD3**	2GV6	Rabbit	Roche	Prediluted	CC1, 36min	20min
**CD20**	L26	Mouse	Roche	Prediluted	CC1, 20min	20min

### Fluorescence *in situ* hybridization

DNA FISH was performed using probes for *HER2*/CEP17, *EGFR*/CEP7 and the break apart *ALK* probe (all from Abbott Laboratories, PathVysion). FISH experiments followed i) a standard protocol as previously described [21]; ii) the standard protocol modified with a preliminary passage in Tris-HCl 0.01 M at pH 8.5 for 3 min at RT before undergoing the standard protocol. Analysis was performed as previously described [21]: 10 invasive areas on each slide were selected and automatically acquired at 40X magnification with the motorized Metafer scanning system (Zeiss, Germany) and Axio Imager epifluorescence microscope. The PathVysion V2 software was used to analyze the results.

### Extraction, quantification and quality assessment of DNA and RNA

Nine sections (5 μm-thick) were obtained from paraffin-embedded tissue blocks of eight colorectal adenocarcinomas processed in parallel, fixed in GAF and in PBF. Sections were deparaffinized using 1 ml of xylene. After over-night incubation at 56°C with proteinase K, DNA was isolated from five sections using the MagCore Genomic DNA FFPE kit on the MagCore automatic extractor instrument (RBC Bioscience, Taiwan) according to manufacturer’s protocol. RNA was obtained using the remaining four sections with RecoverAll Total Nucleic Acid Isolation Kit for FFPE (ThermoFisher Scientific, USA) following the manufacturer’s protocols. Both DNA and RNA extracts were quantified by Qubit BR assay on Qubit Flourometer (Invitrogen, Carlsbad, CA, USA) and NanoDrop Spectophotometer (ThermoFisher Scientific).

DNA and RNA integrity was evaluated with Agilent 2100 Bioanalyzer (Agilent Technologies, USA). DNA integrity was evaluated using High Sensitivity DNA Analysis Kit (Agilent Technologies, Santa Clara, CA) on DNA HS chip. Samples were diluted at 2 ng/μL and DNA length analysis was performed according to manufacturer’s instruction. The average of the DNA fragment size of GAF and PBF samples was assessed using 5000 nt as the threshold for the longer DNA fragments (>5000 nt). Their distribution in respect to this threshold was statistically compared by Chi-square test.

RNA integrity was assessed using Agilent RNA 6000 Nano Kit. The size distribution of the RNA fragments was calculated from Agilent 2100 Bioanalyzer readings using a Smear Analysis with a 200 nt threshold: the percentage of RNA fragments > 200 nt in size (DV200 metric) was recorded [22].

### DNA sequencing analysis

#### Direct sequencing

Fifty ng of DNA was amplified for the exon 2 of *KRAS* (246 bp) using the following PCR condition: 1x buffer, 2.5 mM MgCl2, 0.4 μM of forward and reverse primers (forward: 5’-GGTGGAGTATTTGATAGTGTATTAACC-3’ and reverse: 5’-AGAATGGTCCTG CACCAGTAA-3’), and 0.2 unit of Taq Polymerase in a final volume of 25 μL. PCR reactions were carried out with the following touch-down program: 94°C for 2 minutes, followed by 3 cycles of 94°C for 15 seconds, 64°C for 30 seconds and 70°C for 30 seconds; 3 cycles of 94°C for 15 seconds, 61°C for 30 seconds and 72°C for 30 seconds; 3 cycles of 94°C for 15 seconds, 58°C for 30 seconds and 72°C for 30 seconds; 35 cycles of 94°C for 15 seconds, 57°C for 30 seconds and 72°C for 30 seconds, with a final extension of 70°C for 5 minutes. PCR templates were visualized by electrophoresis on 3% agarose gel, and purified with illustra ExoProStar (GE Healthcare, Italy). A final 15 ng of PCR products were purified with the ExoProStar and used for sequencing analyses. A cycle-sequencing PCR reaction was set up using the Big Dye Version 3.1 Terminator cycle-sequencing kit (ThermoFisher Scientific), with the same amplifying primer added to a final concentration of 5 pmol/μL in a volume of 20 μL. The cycling conditions were: 25 cycles at 96°C for 10 seconds, 50°C for 5 seconds, and 60°C for 4 minutes; the reaction was terminated at 4°C. The cycle sequencing products were purified using Agencourt CleanSEQ (Beckman Coulter, USA), and the DNA was sequenced using an automated 16 capillary sequencer (3730 DNA Analyzer, Applied Biosystems, USA).

#### Pyrosequencing

*KRAS* exon 2 was amplified and sequenced in order to evaluate the status of codon 12 and 13 by pyrosequencing, which is a method based on “sequencing by synthesis” principle, using PSQ 96 (Qiagen, Germany). DNA amplification was performed with the following primers: forward 5’-GGCCTGCTGAAAATCACG-3’, reverse 5’ biotin–GCTCTATCGTCATGGCTCT-3’ (size 80 bp). After denaturation at 94°C for 5 minutes, DNA samples underwent to 40 cycles at 94°C for 45 seconds, 57°C for 45 seconds and 72°C for 1 minute, and a final elongation at 72°C for 5 minutes; the 5’-biotinylated PCR products were bound onto streptavidin-coated paramagnetic beads (GE Healthcare), denaturized by 0.1 mol/l NaOH and released according to the manufacturer’s instructions using PyroMark Vacuum Prep Workstation (Qiagen). These reactions were performed in a 96-wells plate using Pyro Gold Reagents (Qiagen). The primed single-stranded DNA templates were subjected to real-time sequencing of the region including codon 12 and 13 by using the sequencing primer 5’-CTTGTGGTAGTTGTAGCT-3’. The obtained pyrograms were analyzed by using PyroMark ID Software v 1.0 (Qiagen).

#### Mass spectrometry

Samples were analyzed for *KRAS*, *BRAF*, *NRAS* and *PIK3CA* gene mutational status by using Myriapod^®^ Colon status kit (Diatech Pharmacogenetics, Italy). This methodology uses the MassARRAY^®^ strumental system (Sequenom^®^ Inc, USA) based on mass spectrometry MALDI-TOF (Matrix-Assisted Laser Desorption Ionization Time Of Flight) method. DNA samples were first amplified with a multiplex-PCR with Labcycler following the manufacturer’s instructions. Amplified samples were then treated with Shrimp Alkaline Phosphatase (SAP reaction) to remove the excess of nucleotides and primers from PCR reactions. Each purified reaction was then subjected to a single base extension reaction (iPLEX^®^) with modified mass nucleotides. For each polymorphic site, we obtained one or more analytes with a specific mass. Mass spectrometer analysis produces an expected mass peak for each analyte, which will be associated to a wild type or mutant genotype of analyzed sample.

#### Targeted next generation sequencing (NGS)

Samples were subjected to targeted next generation sequencing on an Illumina MiSeq following validated protocols using the Myriapod^®^ NGS-IL 56G Onco-panel (NG032, Diatech Pharmacogenetics), which covers clinically relevant mutational hot-spots of 56 *bona fide* cancer genes (S1 Table).

First, a Real Time PCR that simultaneously amplifies two highly conserved regions, thus generating two PCR products of different length, was used to assess sample quality. The degree of fragmentation was derived from the ratio between the two DNA fragments (larger fragment/shorter fragment) differently labeled with fluorescent probes.

Second, 50 ng of DNA were amplified and processed according to the manufacturers’ instructions. Currently, the protocol does not include a step with uracil-DNA glycosylase (UDG) treatment of DNA samples. Sequencing was performed on an Illumina MiSeq (Illumina^®^ Inc.) and data were processed exploiting the proprietary bioinformatics software associated to Myriapod NGS kits (Diatech Pharmacogenetics).

### RNA analyses

#### cDNA synthesis

A total of 1 μg of RNA was reverse transcribed to cDNA by the Reverse Transcription System Kit (Promega, USA) using 5 mM MgCl2, 1X Buffer, 1 mM dNTPs, 1U/μL Recombinant RNasin Ribonuclease Inhibitor, 1.25U/μL AMV Reverse Transcriptase and a mix of 120 ng of both oligo(dT) and random primers provided by the kit. The cDNA synthesis was carried out incubating at 42°C for 1 h, 95°C for 5 minutes and 2.5°C for 5 minutes. cDNA was quantified with the Qubit ssDNA HS assay kit (ThermoFisher Scientific).

#### Reverse transcriptase-PCR

A total of 50 ng of cDNA was amplified for the exon six of Cytocheratin 20 (*KRT20*) using the following PCR condition: 1x buffer, 2.5 mM MgCl2, 0.4 μM of each primer (forward 5’- AGAGGAGACCAAGGCCCGTTACAG -3’, reverse 5’- CTTCCAGAAGGCGGCGGTAAGTAG -3’) and 0.2 unit of Taq Polymerase in a final volume of 25 μL. PCR reaction was carried out with: 94°C for 3 minutes, followed by10 cycles of 94°C for 15 seconds, 64°C for 30 seconds and 72°C for 30 seconds; 25 cycles of 94°C for 15 seconds, 61°C for 30 seconds and 72°C for 30 seconds with a final extension of 70°C for 5 minutes. PCR templates were visualized by electrophoresis on 3% agarose gel to verify the amplicon size.

## Results

### Stability of the reagents

To overcome the acidification of the fixative the solution here adopted was linked to the addition of ethanol and insoluble calcium carbonate to GAF. This stock solution remained stable for several months. The final (working) solution was obtained by diluting the stock solution 1:10 in 0.11 M phosphate buffer pH 7.3. The resulting 2% GAF in phosphate buffer 0.11 M pH 7.3 (hereafter GAF) is stable for at least one month.

### Structure of cells and tissues

Morphology of the 30 tissues included in the cohort fixed in PBF or in GAF was considered by analyzing nuclear features such as nuclear shape and distribution of chromatin, staining characteristics, shrinkage around glandular structures or cellular aggregates.

**Fig 1:**
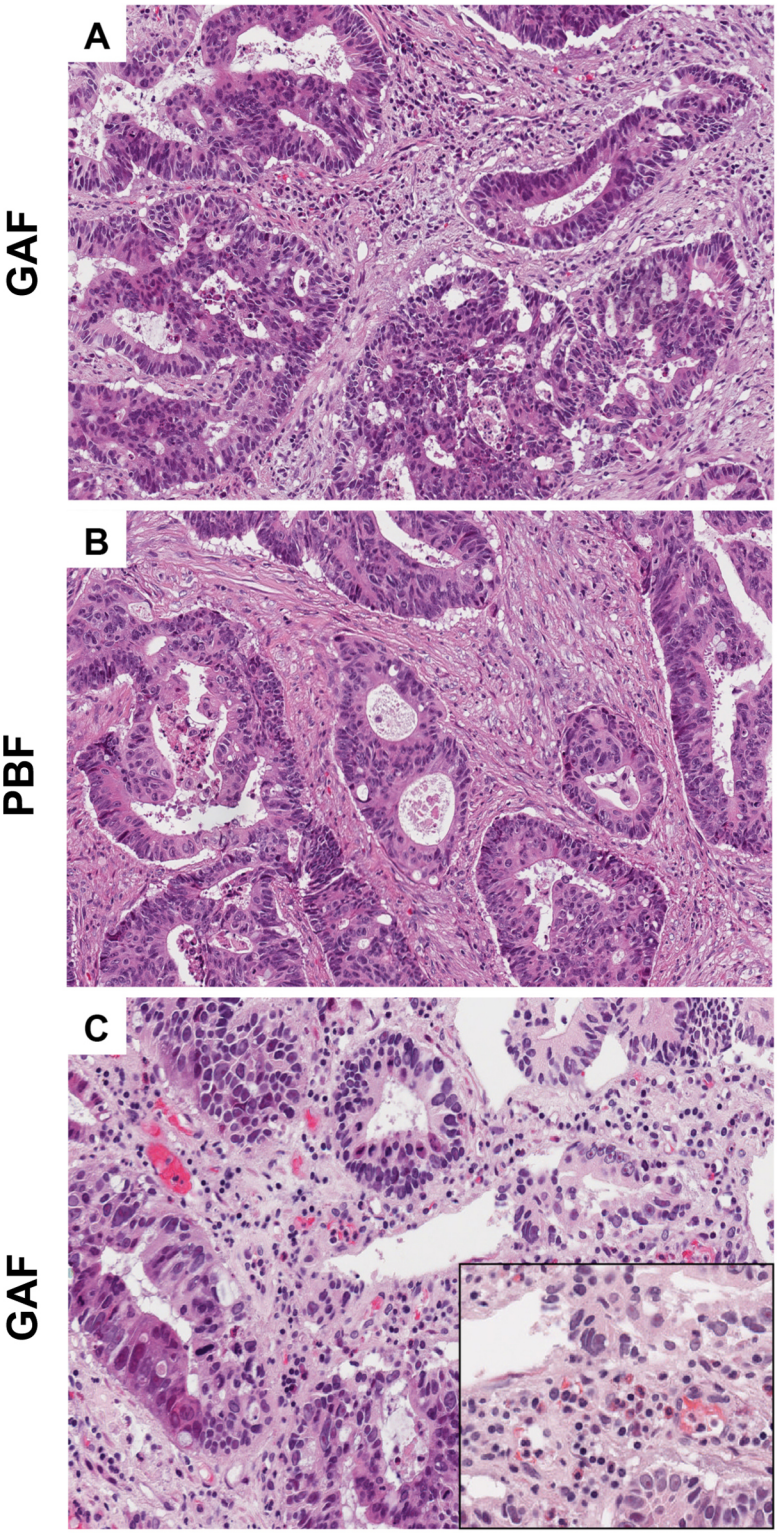
Histology (H&E staining) of tissues fixed either in GAF or in PBF. A colorectal adenocarcinoma fixed in GAF (A, 20X) or in PBF (B, 20X), where the similarity in preservation of structural and cell components can be appreciated. A detailed examination revealed preservation of erythrocytes and no signs of lysis or loss of staining of eosinophils, as shown here in a GAF-fixed colorectal adenocarcinoma (C, 20x and inset, where an area enriched of eosinophils is captured at a higher magnification).

Tissues fixed in commercially available 2% glyoxal in 0.1 M phosphate buffer (an acidic solution) showed lysis of erythrocytes, shrinkage of stromal components, clarity of cytoplasmic components and no evidence of eosinophils. Tissues fixed in GAF presented a degree of structural, cellular and nuclear preservation comparable to that provided by PBF (Fig 1A and 1B, S1 Fig). We observed preservation of erythrocytes and no signs of lysis or of loss of staining of eosinophils (Fig 1C).

### Immunohistochemistry

**Fig 2:**
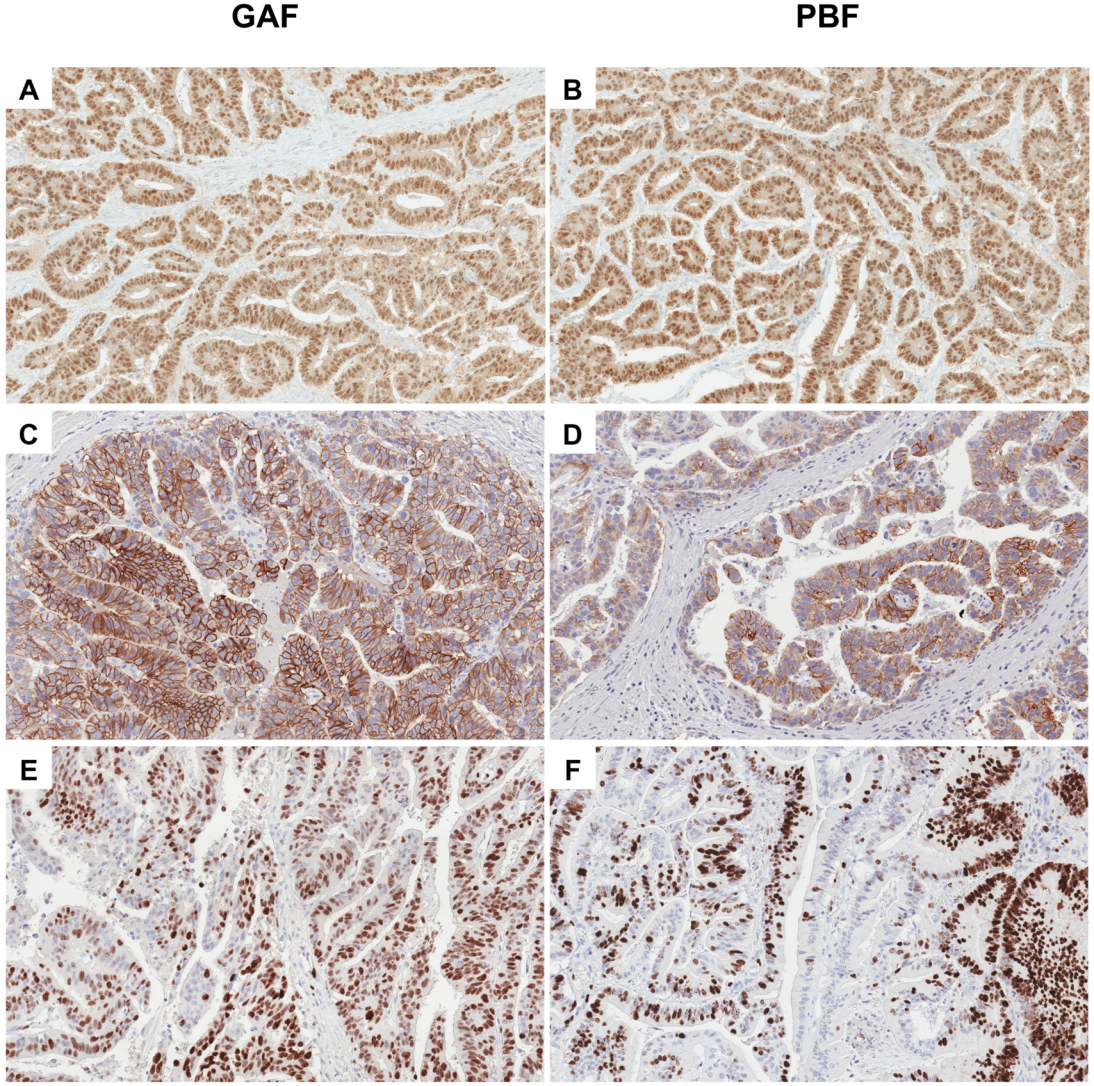
Preservation of antigens in tissues fixed either in GAF or in PBF. (A) CDX2 expression in a GAF-fixed colorectal adenocarcinoma; (B) CDX2 expression in a PBF-fixed sample of the same colorectal adenocarcinoma illustrated in A; (C) HER2 overexpression in a GAF-fixed gastric adenocarcinoma; (D) HER2 overexpression in a PBF-fixed gastric sample of the same adenocarcinoma illustrated in C; (E) proliferation index assessed by Ki67 in a GAF-fixed colorectal adenocarcinoma; (F) proliferation index assessed by Ki67 in a PBF-fixed sample of the same colorectal adenocarcinoma depicted in E.

IHC was employed to assess antigen preservation. The selected target proteins represented a broad spectrum of proteins distributed in different subcellular compartments (cytoplasm, cell membrane, nucleus). No discrepancies in subcellular localization of protein expression were observed in the differently fixed samples (Fig 2). Nuclear antigens required an optimization of the antigen retrieval procedure, i.e. longer duration of the antigen retrieval (60/90 min *versus* 30 min, Table 1). Following optimization, all nuclear antigen except Ki67 gave results superimposable to the reactions performed on PBF fixed samples. Ki67 expression was observed to be less pervasive in GAF fixed samples compared to corresponding PBF samples. A 60 min long antigen retrieval procedure for Ki67 reaction gave better results, however Ki67 indices were lower (mean: 6%) than in formalin fixed samples here analyzed.

For nuclear antigens we also tested antigen retrieval treatment at high temperature (125°C) in a highly basic buffer (pH 8.6). Such treatment did not yield significant improvements compared to longer duration of standard antigen retrieval procedures.

For all of the remaining cytoplasmic and membrane markers, time of antigen retrieval was the same as for standard FFPE tissues.

### Fluorescence *in situ* hybridization analysis

**Fig 3:**
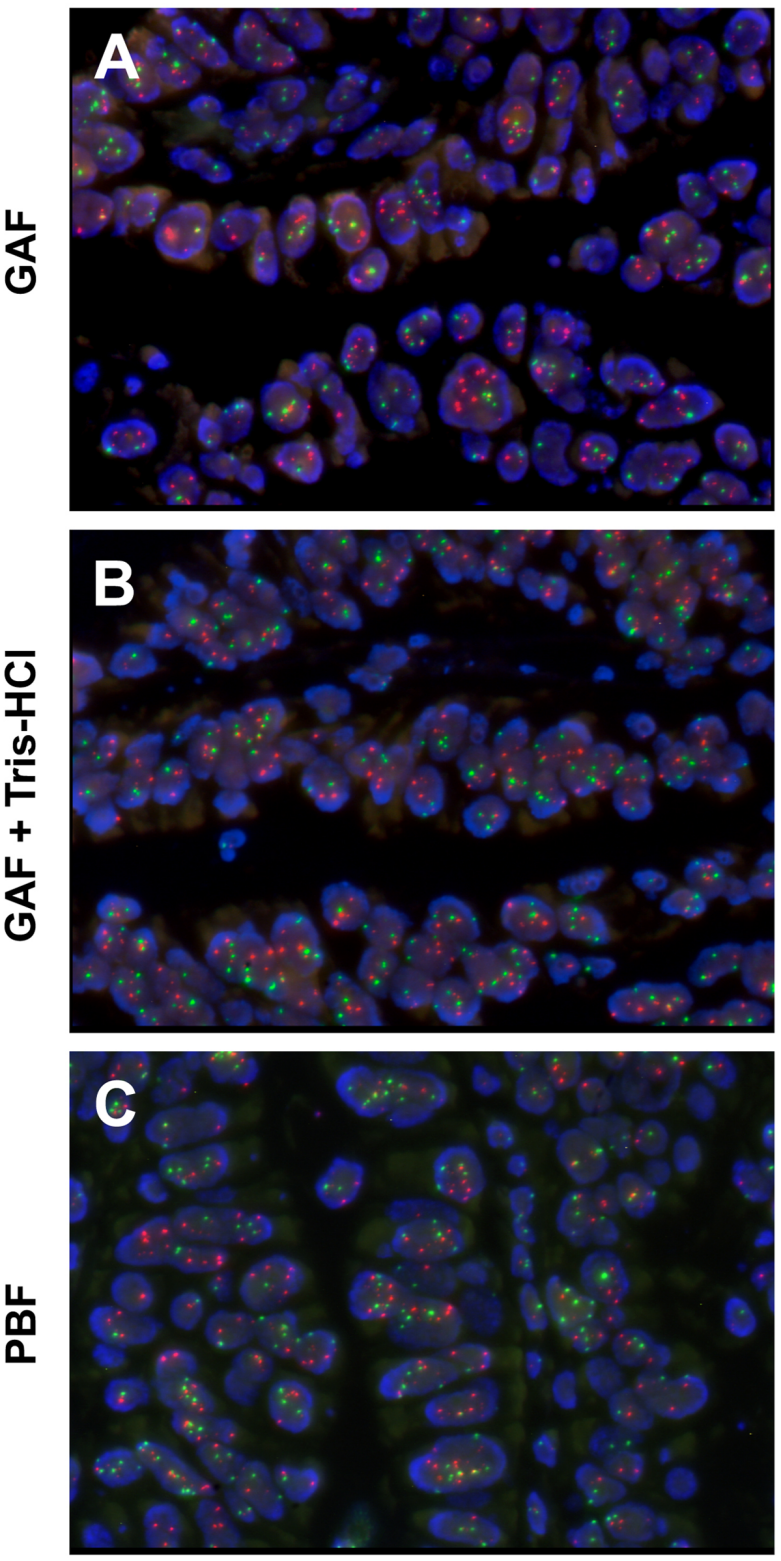
DNA FISH for *HER2* and CEP17 in parallel tissue samples fixed in GAF and in PBF. Representative fields of a breast gastric carcinoma where we performed *HER2*/CEP17 FISH analysis. (A) FISH analysis in a GAF -fixed specimen. (B) The same sample as in A in which sections were treated with a short wash in Tris-HCl 0.01 M at pH 8.5 leading to disappearance of the mild fluorescent background observed in A. (C) Sampling of the same specimen parallel to (A), but fixed in PBF.

FISH analysis following the standard protocol led to a mild autofluorescent background in GAF fixed tissues, which prevented a reliable scoring of signals (Fig 3). The diffuse fluorescence disappeared in sections treated with a short wash in Tris-HCl 0.01 M at pH 8.5 (Fig 3B). Following this pre-treatment, the FISH procedure in the five GAF fixed tissues here analyzed produced results matching those obtained in PBF fixed tissues from the same cases. Mean *HER2* and CEP17 copy numbers, as well as *EGFR* and CEP7 copy numbers were comparable between GAF- and PBF-fixed samples, as per automated scoring by Metafer. Signal intensity was comparable between corresponding samples when pre-treatment with Tris-HCl was performed (Fig 3) and allowed a proper assessment also of fused signals in the experiments using the break apart *ALK* probe.

### DNA sequencing analysis and RNA analysis

**Fig 4:**
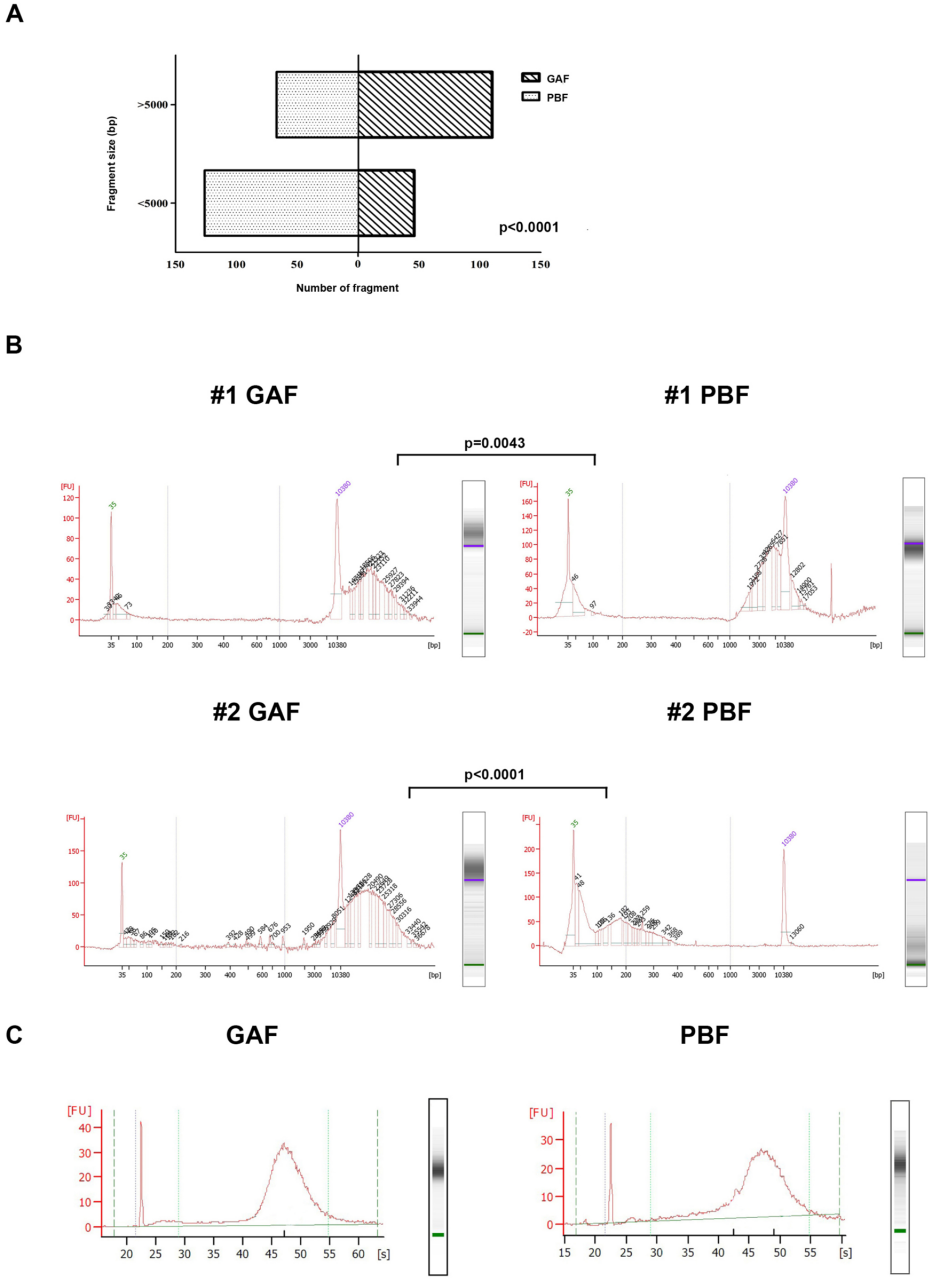
Preservation of nucleic acids in GAF-fixed tissues. Bioanalyzer results of DNA samples extracted from 8 GAF-fixed and matching PFB-fixed colorectal carcinoma specimens. (A) Fragment size distribution of the analyzed DNA samples (threshold for longer fragments: 5000 bp). GAF samples were significantly enriched for less fragmented DNA compared with PBF specimens (Chi-square test). (B) Fragmentation analysis example of two DNA specimens. In both cases the difference of the fragment size average between GAF and PBF samples was statistically significant. (C) RNA quality, determined by Agilent Bioanalyzer traces, was satisfactory in both GAF- and PBF-fixed samples.

To evaluate the preservation of nucleic acids, DNA and RNA were extracted from GAF-fixed and PBF-fixed colorectal adenocarcinomas samples. Agilent Bioanalyzer, providing information about the size range of fragments, was used to assess DNA quality. In terms of DNA fragmentation profile, the fragment size distribution of the 8 analyzed GAF samples was significantly enriched for less fragmented DNA (≥5.000 bp) compared with the matching PBF specimens (p<0.001, Chi-square test, Fig 4A and 4B). These results were corroborated by the Real Time PCR performed as a quality control for targeted NGS: this assay also identified a higher degree of fragmentation in DNA extracted from PBF-fixed samples than in DNA extracted from GAF-fixed samples.

RNA quality, determined by Agilent Bioanalyzer traces, was satisfactory in both PBF- and GAF-fixed samples: the percentage of RNA fragments greater than 200 nt (DV200) was superior to 90% (Fig 4C). The RT-PCR study for *KRT20* expression was carried out in parallel on PBF- and GAF-fixed samples. RNA amplification was successful at 185 bp in parallel samples.

The PBF-fixed samples of the 25 colorectal adenocarcinomas collected in the study had been routinely subjected to Sequenom MassARRAY^®^ to screen for *KRAS/NRAS/BRAF/PIK3CA* mutations. Six samples were found to harbor a *KRAS* codon 12 mutation and two were mutated on *KRAS* codon 117 and 146, respectively. In addition, one sample carried both *KRAS* codon 12 and *PIK3CA* codon 542 mutations. The DNA corresponding to parallel PBF- and GAF-fixed samples of 8 colorectal cancer cases (6 *KRAS* codon 12 mutant and 2 *KRAS* codon 117 and 146 mutant according to the previous test) were then subjected to direct sequencing, pyrosequencing and Sequenom MassARRAY^®^. The pathogenic *KRAS* mutations previously identified in the PBF-fixed specimens were all confirmed in the GAF-fixed samples by direct sequencing, pyrosequencing and Sequenom MassARRAY^®^.

**Fig 5:**
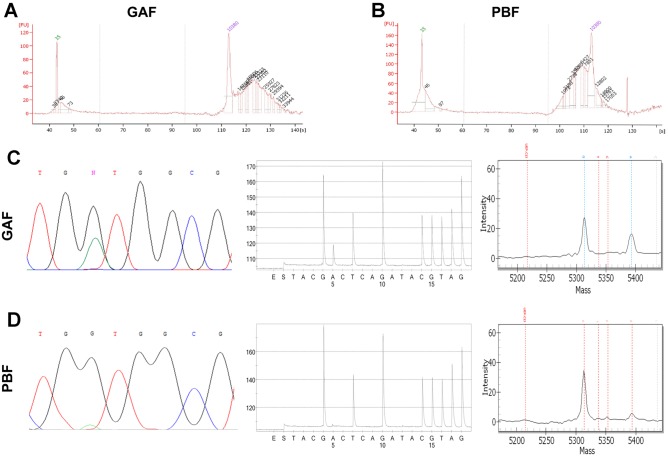
Representative sample pair showing a better DNA preservation in GAF-fixed *versus* PBF-fixed tissues. Representative fragmentation analysis and sequencing results from different methods of two DNA specimens, GAF- and PBF- fixed, respectively (sample pair 4–12). At Bioanalyzer analysis a higher degree of DNA fragmentation was observed in the specimen fixed in PBF (B) compared to the corresponding parallel GAF-fixed sample (A). In addition, the allele frequency of the *KRAS* p.G12D mutation was considerably higher in GAF fixed specimen with all the employed molecular techniques: panel C shows results of the GAF-fixed sample (Direct sequencing, Pyrosequencing, Mass Spectrometry, from left to right), whereas panel D shows corresponding results in the PBF fixed sample (Direct sequencing, Pyrosequencing, Mass Spectrometry, from left to right). Of note, by NGS this mutation showed a mutant allele frequency of 32% in the GAF-fixed sample *versus* 7% in the PBF-fixed sample.

Finally, the eight samples pairs (PBF-fixed and GAF-fixed samples) were subjected to targeted NGS analysis with a panel including 56 *bona fide* cancer genes. The NGS analysis was performed at a comparable mean depth in PBF-fixed (1921X) and GAF-fixed (1934X) samples. Details about the pattern of non-synonymous single nucleotide variants in each sample pair are reported in S2 Fig. The *KRAS/NRAS/BRAF/PIK3CA* mutations detected by Sequenom MassARRAY^®^ were all confirmed in the pairs, however differences in mutant allele frequencies were observed (S2 Fig). Interestingly, in one case (sample pair 4–12) we found that with all the employed molecular techniques, including NGS, the allele frequency of *KRAS* p.G12D mutation was considerably higher in GAF fixed specimen (32.4% vs 7%) (Fig 5). Of note, three pairs (sample pairs 6–14, 7–15, 8–16) presented a series of single nucleotide variants harboring a mutant allele frequency <5%, which were uniquely found in PBF-fixed samples. Formalin fixation can lead to artifactual changes in the DNA double strand [23–25], such as for instance deamination of cytosine to uracil leading to a C:G>T:A change. It is interesting to note that some of these low frequency mutations showed a C:G>T:A call (S2 Fig).

## Discussion

The present study demonstrates that fixation with glyoxal produces a preservation of structural and macro-molecular properties of cells and tissues similar to that obtained by PBF, provided that this non-toxic dialdehyde is used in acid-free conditions (GAF solution).

Previous studies analytically comparing the suitability of glyoxal as a fixative alternative to formalin [3, 12] concluded on the superiority of the latter, given the unsatisfactory histology, unreliable immunohistochemistry and suboptimal nucleic acid preservation in glyoxal-fixed tissues [13–18]. Based on our observations, the reported deleterious effects of glyoxal on the architecture and chemical integrity of tissues are likely linked to the acidity of the fixing solutions. Indeed, commercial solutions of glyoxal are strongly acidic, due to the presence of variable concentrations of glyoxilic, glycolic, formic, acetic and oxalic acids [19]. This leads to the tendency of the dialdehyde to oxidize and progressively acidify in solution, down to pH 4. The main acidic component, i.e. glyoxylic acid, shows a 10 times stronger acidity than acetic acid, with an acid dissociation constant of 4.7 × 10^−4^ (pK_a_ = 3.32). As a consequence, all these acids are bound to exert hydrolytic effects on tissue structure, proteins and nucleic acids. Moreover, previous studies [3, 13, 14, 17, 26, 27] only refer to the use of commercially available formulations having glyoxal as the main component, in addition to other reagents, partly undisclosed. All of these property fixatives (Glyo-Fix, Shandon; Histo-Fix, Bioworld; Histo-CHOICE, Amresco; Prefer, Anatech and Safe-Fix II, Fisher Scientific) are reportedly acidic (in the range of pH 4) and this is likely to represent the reason of the observed drawbacks, especially on nucleic acid preservation.

To guarantee the use of glyoxal in acid-free conditions, we adopted a two-fold procedure. First, a substantial decrease of acids was obtained by the use of ion-exchange resins. Second, long-term (up to 6 months) stable neutral conditions were obtained by the addition of ethanol and calcium carbonate, which is known to react with acids by producing neutral calcium salts and CO_2_.

By using this GAF solution as a histological fixative we obtained a satisfactory preservation of tissue and cell components. Specifically, unlike what previously reported [12, 13] erythrocytes and eosinophils were properly stained. Immunohistochemical tests performed in GAF-fixed material gave results basically matching the tests performed on PBF-fixed on parallel tissue blocks.

Of note, the present results on antigen detection by IHC are different from those reported by previous groups [12–14] using acidic glyoxal solutions such as Prefer or Glyo-Fix, where the immuno-histochemical results were generally regarded as sub-optimal. It has been reported that some antigens may require treatment at high temperature (125°C) in a highly basic buffer (pH 8,6) [12]. Such treatment was not needed in our material and, when tested, did not yield significant improvement. We should point out however that Ki67 staining gave satisfactory results only following a longer treatment (60’ *versus* 30’) using an acidic buffer. Under these conditions and in the limited number of samples tested, the intensity of the staining was satisfactory, however the number of Ki67 positive nuclei was lower than those detectable in the parallel PBF-fixed tissues. More extensive, detailed and comparative studies, also including systematic automatic scoring of Ki67 reactions are warranted to evaluate quantitative differences between GAF- and PBF-fixed tissues as this may have an impact in subclassification of lesions and their related malignant potential.

In terms of molecular pathology analyses, the mild autofluorescent background with FISH testing is likely due to the link of glyoxal to the DNA bases, mainly to guanine and cytidine [28], which results in cross-links most likely responsible of the observed nuclear auto-fluorescence. In line with previous observations by Hutton and Wetmur [29] on the reversibility of the binding of glyoxal to DNA in alkaline pH, the autofluorescence disappeared following a short passage in an alkaline buffer (pH 8.6).

Finally, the three sequencing platforms here tested (Sanger sequencing, Pyrosequencing, Sequenom MassARRAY^®^) gave comparable results for *KRAS* testing on both PBF- and GAF-fixed samples suggesting a DNA/RNA fragmentation not lower than that induced by formalin crosslinking. Of note, we observed an enrichment of the GAF-fixed samples for longer DNA fragment size. In our study, the performance of the NGS analysis was comparable between GAF-fixed ad PBF-fixed samples, nevertheless the platform here employed relied on a robust targeted NGS assay designed for DNA extracted from FFPE samples. One could hypothesize that whole exome sequencing approaches are likely to yield better results in GAF-fixed samples showing a lesser degree of fragmentation/degradation. It is interesting to note that some mutations detected at low mutant allele frequency (<5%) in PBF-fixed samples showed the typical C:G>T:A call, which may represent an artifactual change most readily explained as a consequence of cytosine being deaminated to uracil, a known consequence of formalin fixation [23–25]. Larger studies are warranted to ascertain whether GAF fixed samples could reliably minimize C:G>T:A changes as well as other artefactual calls induced by formalin fixation.

In conclusion, the data here presented indicate that fixation in a pH 7.2–7.4 GAF solution results in a histological, immunohistochemical and nucleic acid preservation not inferior to that permitted by fixation in PBF. Importantly, DNA appears to be better preserved by GAF rather than by PBF fixation.

Substitution of formalin as the reference fixative in histopathology is recommended because of its toxicity, but cannot be presently practiced since this might jeopardize the invaluable array of common morphological, immunophenotypic and molecular parameters currently derived from PBF-fixed tissues and playing a pivotal role in personalized therapies.

Adoption of GAF as a non-toxic histological fixative of choice would therefore require a process of validation, but the present study indicates that it represents a reliable candidate.

## Supporting information

S1 Fig Parallel tissue samples, fixed in GAF and PBF, of normal colonic mucosa showing comparable preservation of tissue architecture. (TIF)

S2 Fig Non synonymous single nucleotide variants (SNVs) detected by using the Myriapod^®^ NGS-IL 56G Onco-panel in each sample pair (GAF- and corresponding PBF-fixed samples for the eight cases subjected to molecular assays). Ranges of mutant allele frequencies are color-coded according to the legend on the right hand side. Squares with black borders identify those variants with a mutant allele frequency (MAF) <5% showing a C:G>T:A call.
(PDF)

S1 Table List of genes represented in Myriapod^®^ NGS-IL 56G Onco-panel (NG032, Diatech Pharmacogenetics, Jesi, Italy) and number of amplicons. (DOCX)
