# Supplementary figures and images for: Acid-free glyoxal as a substitute of formalin for structural and molecular preservation in tissue samples
Source: PLoS One. 2017 Aug 10;12(8):e0182965. doi: 10.1371/journal.pone.0182965 (PMC5552132; doi:10.1371/journal.pone.0182965)

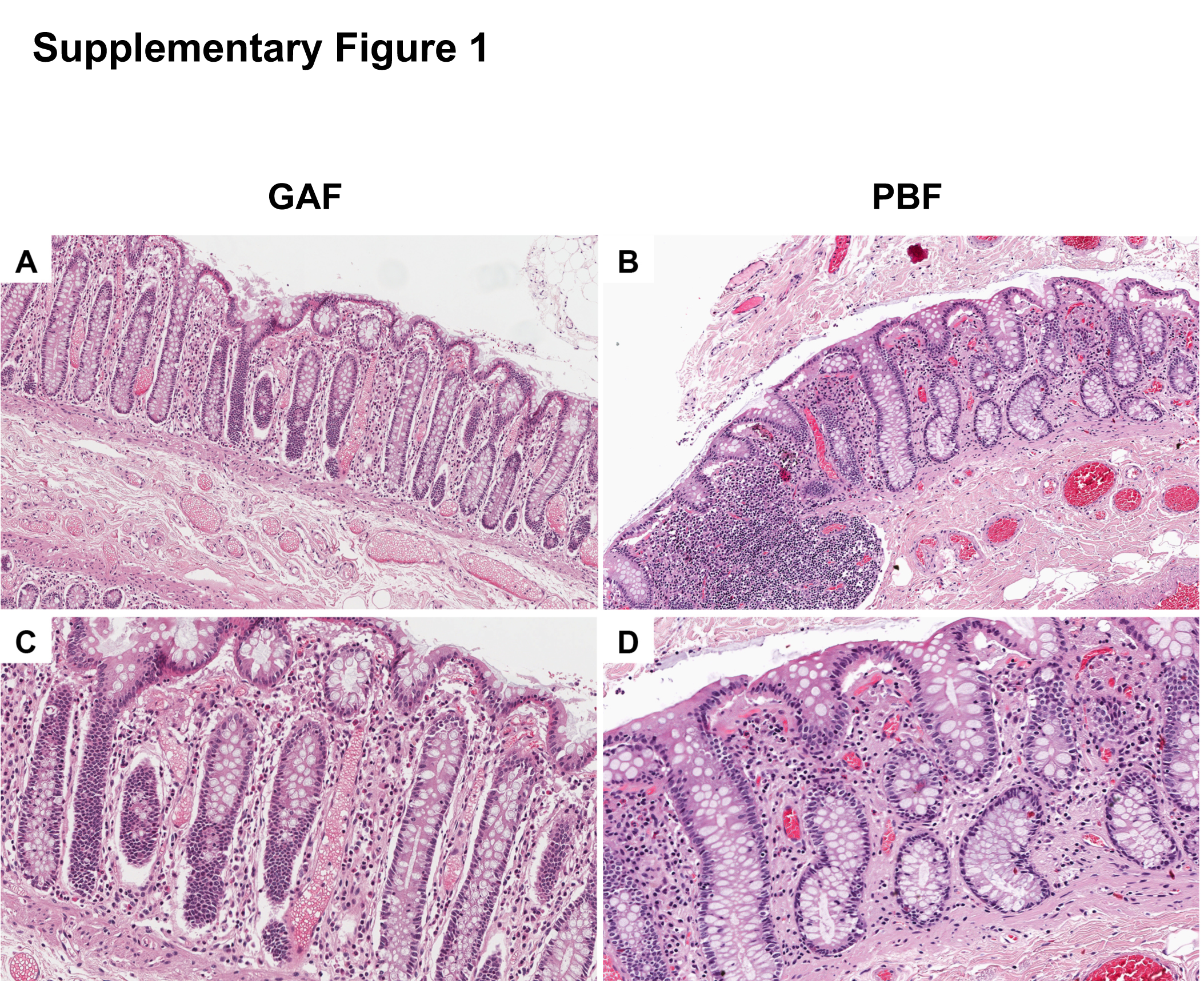

Supplement: S1 Fig — (TIF) [file pone.0182965.s001.tif]

# Supplementary Figure 1

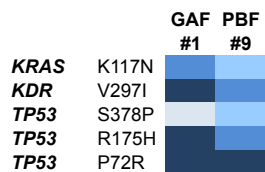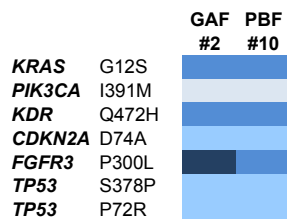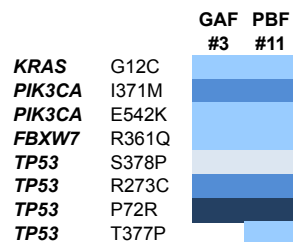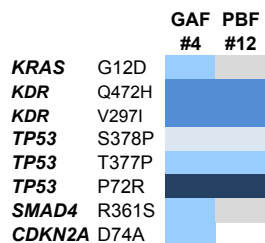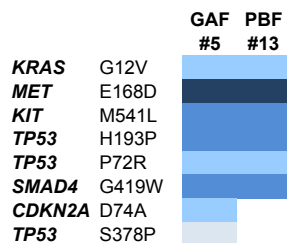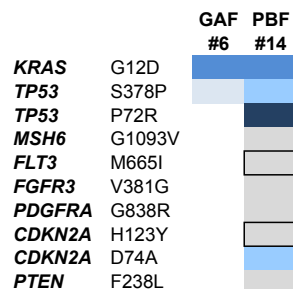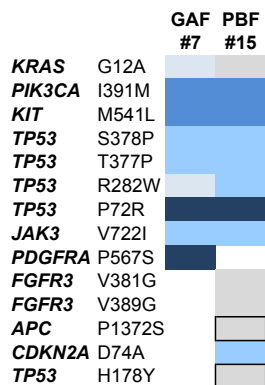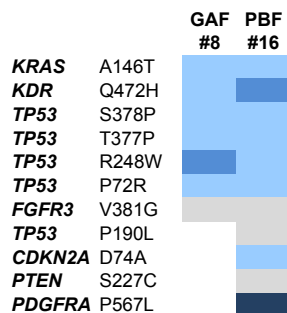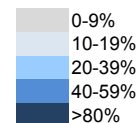

□ C:G>T:A change in a SNV with a MAF<5%

Supplement: S2 Fig — Ranges of mutant allele frequencies are color-coded according to the legend on the right hand side. Squares with black borders identify those variants with a mutant allele frequency (MAF) <5% showing a C:G>T:A call. (PDF) [file pone.0182965.s002.pdf]
