# Supplementary material for: Acid-free glyoxal as a substitute of formalin for structural and molecular preservation in tissue samples
Source: PLoS One. 2017 Aug 10;12(8):e0182965. doi: 10.1371/journal.pone.0182965 (PMC5552132; doi:10.1371/journal.pone.0182965)
Supplement: S1 Table — (DOCX) [file pone.0182965.s003.docx]

**Supplementary Table 1: List of genes represented in Myriapod® NGS-IL 56G Onco-panel (NG032, Diatech Pharmacogenetics, Jesi, Italy) and number of amplicons.**

| **N** | **Gene name** | **Amplicons** |
| --- | --- | --- |
| 1 | *ABL1* | 5 |
| 2 | *AKT1* | 2 |
| 3 | *ALK* | 2 |
| 4 | *APC* | 9 |
| 5 | *ATM* | 19 |
| 6 | *BRAF* | 2 |
| 7 | *CDH1* | 3 |
| 8 | *CDKN2A* | 2 |
| 9 | *CSF1R* | 2 |
| 10 | *CTNNB1* | 1 |
| 11 | *DDR2* | 1 |
| 12 | *DNMT3A* | 1 |
| 13 | *EGFR* | 9 |
| 14 | *ERBB2* | 4 |
| 15 | *ERBB4* | 8 |
| 16 | *EZH2* | 1 |
| 17 | *FBXW7* | 6 |
| 18 | *FGFR1* | 2 |
| 19 | *FGFR2* | 4 |
| 20 | *FGFR3* | 6 |
| 21 | *FLT3* | 4 |
| 22 | *FOXL2* | 1 |
| 23 | *GNA11* | 2 |
| 24 | *GNAQ* | 2 |
| 25 | *GNAS* | 2 |
| 26 | *HNF1A* | 4 |
| 27 | *HRAS* | 2 |
| 28 | *IDH1* | 1 |
| 29 | *IDH2* | 2 |
| 30 | *JAK2* | 2 |
| 31 | *JAK3* | 3 |
| 32 | *KDR* | 9 |
| 33 | *KIT* | 14 |
| 34 | *KRAS* | 3 |
| 35 | *MAP2K1* | 5 |
| 36 | *MET* | 6 |
| 37 | *MLH1* | 1 |
| 38 | *MPL* | 1 |
| 39 | *MSH6* | 4 |
| 40 | *NOTCH1* | 3 |
| 41 | *NPM1* | 1 |
| 42 | *NRAS* | 3 |
| 43 | *PDGFRA* | 4 |
| 44 | *PIK3CA* | 11 |
| 45 | *PTEN* | 14 |
| 46 | *PTPN11* | 2 |
| 47 | *RB1* | 12 |
| 48 | *RET* | 6 |
| 49 | *STK11* | 5 |
| 50 | *SMAD4* | 10 |
| 51 | *SMARCB1* | 4 |
| 52 | *SMO* | 5 |
| 53 | *SRC* | 1 |
| 54 | *TP53* | 21 |
| 55 | *TSC1* | 1 |
| 56 | *VHL* | 3 |
